# Addressing symptoms that affect patients’ eating according to the Head and Neck Patient Symptom Checklist^©^

**DOI:** 10.1007/s00520-022-07038-x

**Published:** 2022-04-15

**Authors:** Brith Granström, Thorbjörn Holmlund, Göran Laurell, Per Fransson, Ylva Tiblom Ehrsson

**Affiliations:** 1 Department of Clinical Science, Otorhinolaryngology, Umeå University, 901 87 Umeå, Sweden; 2 Department of Surgical Sciences, Section of Otorhinolaryngology and Head & Neck Surgery, Uppsala University, 751 85 Uppsala, Sweden; 3 Department of Nursing, Umeå University, 901 87 Umeå, Sweden

**Keywords:** Head and neck cancer, Nutritional impact symptoms, Body weight loss, Health-related quality of life, Head and Neck Patient Symptom Checklist^©^

## Abstract

**Purpose:**

The purpose of this prospective study was to assess which nutritional impact symptoms (NIS) interfere with oral intake in patients with head and neck cancer (HNC) and how the symptoms interfere with body weight loss, up to 1 year after treatment.

**Methods:**

This was a prospective study of 197 patients with HNC planned for treatment with curative intention. Body weight was measured before the start of treatment, at 7 weeks after the start of treatment, and at 6 and 12 months after completion of treatment. NIS and NIS interfering with oral intake at each follow-up were examined with the Head and Neck Patient Symptom Checklist^©^ (HNSC^©^).

**Results:**

At 7 weeks of follow-up, patients experienced the greatest symptom and interference burden, and 12 months after treatment the NIS scorings had not returned to baseline. One year after treatment, the highest scored NIS to interfere with oral intake was swallowing problems, chewing difficulties, and loss of appetite. At all 3 follow-ups, the total cumulative NIS and NIS interfering with oral intake were associated with body weight loss. Factors increasing the risk for a body weight loss of ≥ 10% at 12 months after treatment were pain, loss of appetite, feeling full, sore mouth, difficulty swallowing, taste changes, and dry mouth. Women scored higher than men in NIS and NIS interfering with oral intake. Furthermore, during the study period about half of the population had a body weight loss > 5%.

**Conclusion:**

Because both nutritional and clinical factors may affect body weight, this study highlights the importance of a holistic approach when addressing the patients’ nutritional issues.

**Trial registration:**

ClinicalTrials.gov NCT03343236, date of registration: November 17, 2017.

## Introduction

Patients with head and neck cancer (HNC) are at pronounced risk for body weight loss due to tumour burden [1] and treatment [2]. Weight loss often indicates a frail nutritional status and reduced health-related quality of life (HRQoL) [3]. Over time, some patients develop cancer-induced cachexia, which is a paraneoplastic syndrome characterized by muscle wasting that is reported to be a risk factor for poor treatment outcome [4].

The main treatments for HNC are radiotherapy (RT) and surgery as single modality treatments or in combination. In addition to RT, chemotherapy or immunotherapy can be given. During treatment body weight tend to decrease and approximately 15–26% of the patients with HNC demonstrate a body weight loss > 10% at the end of treatment [5, 6]. Weight loss also tends to continue for several months after treatment before recovery is notable [1, 7, 8]. Several individual and disease-related variables are recognized as risk factors for weight loss, such as tumour stage [9], tumour site, pre-treatment high body mass index [9, 10], and systemic inflammation [11, 12]. Furthermore, RT and chemoradiation treatment-related side effects that may affect oral intake — such as sore mouth, depression, swallowing difficulties [13], sticky saliva, fatigue [14], and trismus [15] — increase during RT and chemoradiation and are most pronounced at the end of treatment [16]. Still, several symptoms such as loss of appetite, fatigue, dry mouth, sticky saliva, coughing, and dental issues may remain 1-year post-treatment [17]. Research investigating any potential relation between the severity of symptom burden and weight loss is sparse. Lee (2019) reported findings concerning overall symptom burden and weight loss during the treatment period [5], and greater weight loss related to eating difficulties has been described up to a couple of months after treatment [13] and at 12 months after treatment [18].

The EORTC QLQ-C30 [19] and the HNC-specific EORTC QLQ-H&N 35 [20] are frequently used HRQoL questionnaires in research but less so in clinical settings. These two questionnaires cover a wide range of topics and have been extensively used to investigate the effects of HNC and treatment on HRQoL. The Head and Neck Patient Symptom Checklist^©^ (HNSC^©^) [13, 21] is constructed to especially identify nutritional impact symptoms (NIS) and to what degree these symptoms affect oral intake. Previous and resent research using the HNSC^©^ has described the relation between NIS burden and weight loss, with NIS burden before treatment being a predictor for weight loss, nutritional intake, and survival [22]. Increasing NIS burden during the treatment period is reported to negatively affect body weight [13, 23–25] and increase the need for oral nutritional supplements [24]. To our knowledge, Kubrak et al. (2013) is the only study reporting NIS-weight loss pattern up to 2.5 months after treatment using the HNSC^©^ [13]. Because treatment-related side effects may be of both acute and long-lasting character, NIS need to be identified early and followed-up by health care professionals. Thus, research on NIS burden and its interference with oral intake over longer periods of time are needed. The purpose of this prospective study was to assess which NIS interfere with oral intake in patients with HNC and how the symptoms interfere with body weight loss up to 1 year after treatment.

## Materials and methods

### Study population

This study was a planned sub-study of a larger prospective, observational, multicentre research study performed at three university hospitals in Sweden (ClinicalTrials.gov NCT03343236). Patients referred to any of the three hospitals were asked for participation before the start of treatment and were thereafter enrolled. Patients were eligible for inclusion if they were over 18 years of age, had a histologically confirmed HNC, were planned for treatment with curative intention, and had performance status 0–2 according to the Eastern Cooperative Oncology Group Performance Status/World Health Organization Performance Status (WHO PS, with the categories of 0 “fully active”, 1 “restricted in physically strenuous activity but ambulatory and able to carry out work”, 2 “ambulatory and capable of all self-care but unable to carry out any work activities”, 3 “capable of only limited self-care”, 4 “completely disabled”, and 5 “dead”) [26] at the time of diagnosis. Exclusion criteria were treatment for a malignant disease within the last 5 years, severe alcoholism/drug abuse, cognitive impairments, and lack of understanding of the Swedish language. All three hospitals followed the national guidelines for treatment and nutritional support. All patients were under nutritional surveillance and were offered nutritional treatment when needed. A body weight loss >5% of pre-treatment weight, an advanced staged tumour (IV), and/or expected nutritional difficulties were the basis for tube feeding.

### Data collection

Between October 2015 and March 2018, 220 patients accepted participation. Eligible for the present study were patients who had completed the follow-up at 7 weeks, leaving 197 included in this study. Clinical characteristics are presented in Table 1. Baseline measurements were performed adjacent to initiation of treatment, at follow-up at 7 weeks after the start of treatment, and then at 6 and 12 months after the completion of treatment. The number of participants decreased during the study period from 197 to 184 patients at the 6-month follow-up (9 patients were deceased or too ill to continue the study and 4 patients chose to drop out) and to 177 patients at the 12-month follow-up (a further 6 patients were deceased or too ill to continue the study, and 1 patient suffered from cognitive dysfunction). Some of the follow-ups were performed at the local hospital or, due to long travel distance, via telephone at 6 and 12 months (*n* = 114 and *n* = 95, respectively). For data management and for facilitating data collection and access, a web-based reporting system (data.dynareg.se) was developed for the research study. Body weight was measured on all occasions, and when the follow-ups were held via telephone the patients used their own scales, and the patients filled in the HNSC^©^. The patients could choose to answer the questionnaire as web-based or on paper. If a paper questionnaire was used, the research nurse transferred the answers into the web-based program. On all four occasions, the HNSC^©^ contained missing values because some patients chose to leave the answer blank. Medical data such as tumour site, stage, and treatment were obtained from the patient’s medical records. All participants received oral and written information, and written consent was obtained. This study was approved by the Regional Ethical Review Board in Uppsala (No. 2014/447).

**Table 1:** Clinical characteristics of 197 patients with head and neck cancer. Numbers and percentages are given, *n* (%)

Characteristics	*n* (%)
Age, years, mean ± SD (min–max)	63 ± 11 (32–89)
Age, years	
< 70 ≥ 70	137 (70) 60 (30)
Gender	
Male Female	144 (73) 53 (27)
Tumour site	
Oropharynx Oral cavity Larynx Other*	89 (45) 57 (29) 24 (12) 27 (14)
Tumour stage, UICC 8†	
I-II III-IV	121 (61) 76 (39)
Treatment	
Surgery Radiotherapy ± surgery‡ Radiotherapy + chemo- or other pharmacological treatment ± surgery ≠	24 (12) 104 (53) 69 (35)

^*^Hypopharynx (*n* = 6), cancer of unknown primary (*n* = 6), salivary gland cancer (*n* = 5), nasal or sinus cancer (*n* = 5), nasopharynx (*n* = 3), and cancer involving the external auditory canal (*n* = 2). †Union for International Cancer Control 8th edition. ‡Seven patients received radiotherapy + brachytherapy, and 61/104 patients had surgery. ≠ Fifty-one patients received cisplatin, 1 patient received carboplatin, 17 patients received cetuximab, and 24/69 patients had surgery

The HNSC^©^ is designed and validated for patients with HNC [13, 21] and was used in this study with the kind permission of Springer Science + Business Media. The questionnaire measures patients’ perceived NIS and whether and to what extent the symptoms had interfered with oral intake during the past 3 days. The form consists of questions concerning 17 symptoms and the patient has to answer “how often did you have this symptom” followed-up by the question “has this symptom interfered with eating”. The answers are graded on a Likert scale from 1 (not at all) to 5 (a lot), meaning a total symptom score ranging from 17 (no symptoms) to 85 (scoring 5 in all 17 symptoms). Finally, patients can add additional symptoms in writing [15] (not presented in this study). For this project, the HNSC^©^ was translated into Swedish using “Translation and Cultural Adaption of Patient Reported Outcomes Measures—Principles of Good Practice” [27].

### Statistical analysis

Patient demographic data and clinical characteristics are presented as the mean and standard deviation (SD). To analyse the number of patients scoring NIS and NIS interfering with oral intake, scores of 1–5 using the HNSC^©^ were included in the analysis and summarized as the mean (SD). A total sum score for NIS and NIS interfering with oral intake was calculated by calculating the sum of all 17 NIS scores, ranging from 17 to 85. A multiple linear regression model was used to model the relationship between NIS and NIS interfering with oral intake and the clinical parameters of gender, age, stage, treatment, and localisation. In the regression models, the score variable was dependent and the clinical parameters were independent. The normality assumption was assessed by studying the residuals with histograms and Q-Q plots. The homoscedasticity assumption was checked by plotting the fitted values against the residuals. For each time point, the patient’s cumulative NIS and NIS interfering with oral intake were calculated as the mean value of each patients’ corresponding ratings reported up to that time point. For example, a patient’s cumulative pain at 6 months was calculated as the mean of the pain ratings at baseline, 7 weeks, and 6 months. This was done for each of 17 NIS which are displayed in a forest plot, as well as for the total NIS sum index and for the total NIS interfering with oral intake sum index. The relation between percentage weight loss and cumulative NIS was estimated using a mixed-models repeated measures (MMRM) model, with percentage weight loss as the dependent variable. Independent variables were timepoint (7 weeks, 6, and 12 months) and cumulative NIS as a continuous variable, including an interaction between time point and cumulative NIS. An unstructured covariance matrix was used for the model error term. The slope for the cumulative NIS at each time point was estimated by combining the NIS and NIS × Time interaction coefficients using the function *emtrends* in the R package *emmeans*, from which *p*-values and 95% confidence intervals were also inferred [28]*.* The linearity assumption between percentage weight loss and cumulative NIS in the MMRM was assessed by refitting corresponding models while modelling the effect of NIS using restricted cubic splines with three knots at the 10th, 50th, and 90th percentiles. The spline models were thereafter compared to the original models using log-likelihood ratio tests. A non-significant test was interpreted to mean that use of nonlinear effects did not improve the model fit. Logistic regression models adjusted for the effect of age, gender, stage, treatment, and localisation were used to evaluate the association between weight loss ≥ 10% at the 12-month follow-up and NIS and NIS interfering with oral intake. Pearson’s chi-squared or Fisher’s exact tests were used for analysing experienced NIS and NIS interfering with oral intake at the 12-month follow-up in patients with a body weight loss < 10% or ≥ 10%. The NIS and NIS interference scores were dichotomised as 1 = no symptom/interference and 2 = symptom/interference scores of 2–5. Statistical analyses were performed using IBM SPSS version 26.0 (IBM, Armonk, NY, USA). The regression analysis and the figures were produced using R (R v4.0.3, R Core Team). All significance levels were set to *p* < 0.05 and all tests were two-tailed.

## Results

### NIS and NIS interference with oral intake

Table 2 shows NIS mean levels of symptom burden at baseline (range 1.0–2.2), 7 weeks (range 1.5–3.7), 6 months (range 1.1–3.1), and 12 months (range 1.1–3.0). The highest NIS scores at baseline were seen for pain, anxiety, and sore mouth. At the 12-month follow-up, the highest NIS scores were found for dry mouth, thick saliva, and taste changes. Table 3 shows the mean scores for NIS interfering with oral intake at baseline (range 1.5–2.7), 7 weeks (range 1.8–3.5), 6 months (range 1.7–2.7), and 12 months (range 1.3–2.6). At baseline, the highest NIS interfering with oral intake scores were in swallowing and chewing difficulties, sore mouth, and loss of appetite. At the 12-month follow-up, the highest NIS interference scores were in swallowing and chewing difficulties and loss of appetite.

**Table 2:** The number of patients with head and neck cancer reporting nutritional impact symptoms (NIS) during the past 3 days using the Head and Neck Patient Symptom Checklist^©^ (HNSC^©^). Mean values ± standard deviation (SD) of severity score 1–5

	Baseline, adjacent to initiation of treatment. Total = 197		Follow-up 7 weeks after start treatment. Total = 197		Follow-up 6 months after completion of treatment. Total = 184		Follow-up 12 months after completion of treatment. Total = 177	
Total	*n* (%)	Mean ± SD	*n* (%)	Mean ± SD	*n* (%)	Mean ± SD	*n* (%)	Mean ± SD
Pain	190 (96)	2.2 ± 1.2	175 (89)	3.3 ± 1.3	169 (92)	2.0 ± 1.1	166 (94)	1.8 ± 1.1
Anxious	189 (96)	2.2 ± 1.1	174 (88)	1.9 ± 1.0	169 (92)	1.7 ± 1.0	165 (93)	1.8 ± 1.0
Thick saliva	189 (96)	1.8 ± 1.1	175 (89)	3.5 ± 1.3	168 (91)	2.4 ± 1.4	164 (93)	2.4 ± 1.3
Dry mouth	189 (96)	1.8 ± 1.1	174 (88)	3.2 ± 1.3	169 (92)	3.1 ± 1.3	164 (93)	3.0 ± 1.4
Loss of appetite	189 (96)	1.6 ± 1.0	175 (89)	3.0 ± 1.5	169 (92)	1.9 ± 1.1	164 (93)	1.8 ± 1.1
Constipation	189 (96)	1.4 ± 0.9	174 (88)	2.0 ± 1.3	167 (91)	1.4 ± 0.9	165 (93)	1.5 ± 1.0
Diarrhoea	189 (96)	1.3 ± 0.6	174 (88)	1.5 ± 1.0	169 (92)	1.3 ± 0.7	165 (93)	1.2 ± 0.6
Sore mouth	188 (95)	2.0 ± 1.2	174 (88)	3.1 ± 1.5	168 (91)	1.9 ± 1.1	166 (94)	1.7 ± 1.1
Lack of energy	188 (95)	1.8 ± 1.0	174 (88)	2.9 ± 1.3	166 (90)	2.2 ± 1.1	165 (93)	2.0 ± 1.1
Feeling full	188 (95)	1.9 ± 1.1	174 (88)	2.3 ± 1.3	166 (90)	2.1 ± 1.1	165 (93)	1.9 ± 1.1
Vomiting	188 (95)	1.0 ± 0.2	174 (88)	1.6 ± 1.1	169 (92)	1.1 ± 0.3	165 (93)	1.1 ± 0.5
Depressed	188 (95)	1.5 ± 0.7	174 (88)	1.6 ± 0.9	169 (92)	1.5 ± 0.8	165 (93)	1.4 ± 0.8
Difficulty chewing	187 (95)	1.7 ± 1.1	175 (89)	2.7 ± 1.5	169 (92)	2.0 ± 1.3	166 (94)	1.8 ± 1.2
Difficulty swallowing	187 (95)	1.9 ± 1.2	175 (89)	3.1 ± 1.5	168 (91)	2.0 ± 1.2	163 (92)	1.9 ± 1.2
Taste changes	187 (95)	1.5 ± 1.1	175 (89)	3.7 ± 1.4	169 (92)	2.5 ± 1.3	166 (94)	2.4 ± 1.3
Nausea	187 (95)	1.2 ± 0.6	175 (89)	1.8 ± 1.2	167 (91)	1.2 ± 0.5	166 (94)	1.2 ± 0.6
Smells bother me	187 (95)	1.2 ± 0.7	173 (88)	1.9 ± 1.3	167 (91)	1.4 ± 0.8	166 (94)	1.4 ± 0.8

**Table 3:** The number of patients with head and neck cancer reporting nutritional impact symptoms (NIS) interfering with oral intake during the past 3 days using the Head and Neck Patient Symptom Checklist^©^ (HNSC^©^). Mean values ± standard deviation (SD) of severity score 1–5

	Baseline, adjacent to initiation of treatment. Total = 197		Follow-up 7 weeks after start treatment. Total = 197		Follow-up 6 months after completion of treatment. Total = 184		Follow-up 12 months after completion of treatment. Total = 177	
Total *n*	*n* (%)	Mean ± SD	*n* (%)	Mean ± SD	*n* (%)	Mean ± SD	*n* (%)	Mean ± SD
Pain	121 (61)	2.4 ± 1.2	156 (79)	3.5 ± 1.4	89 (48)	2.3 ± 1.2	76 (43)	2.3 ± 1.2
Sore mouth	96 (49)	2.6 ± 1.2	132 (67)	3.4 ± 1.3	80 (43)	2.3 ± 1.2	65 (37)	2.2 ± 1.0
Difficulty swallowing	81 (41)	2.7 ± 1.1	137 (70)	3.4 ± 1.4	83 (45)	2.6 ± 1.1	83 (47)	2.5 ± 1.2
Difficulty chewing	70 (36)	2.7 ± 1.1	112 (57)	3.3 ± 1.3	79 (43)	2.7 ± 1.2	68 (38)	2.6 ± 1.1
Loss of appetite	67 (34)	2.6 ± 1.0	135 (69)	3.3 ± 1.3	82 (45)	2.6 ± 1.0	71 (40)	2.5 ± 1.0
Feeling full	81 (41)	2.2 ± 1.0	108 (55)	2.6 ± 1.2	101 (55)	2.3 ± 1.1	82 (46)	2.2 ± 1.0
Anxious	126 (64)	1.7 ± 1.0	95 (48)	2.1 ± 1.1	67 (36)	1.9 ± 0.9	70 (40)	1.8 ± 0.9
Dry mouth	79 (40)	1.9 ± 1.0	142 (72)	2.7 ± 1.4	137 (74)	2.3 ± 1.2	127 (72)	2.3 ± 1.2
Depressed	67 (34)	1.8 ± 0.8	63 (32)	2.1 ± 1.0	53 (29)	1.7 ± 0.8	41 (23)	1.8 ± 1.0
Thick saliva	80 (41)	1.7 ± 1.1	155 (79)	2.7 ± 1.4	104 (57)	2.2 ± 1.2	109 (62)	1.9 ± 1.0
Lack of energy	88 (45)	1.6 ± 1.0	142 (72)	2.3 ± 1.2	102 (55)	1.8 ± 1.1	93 (53)	1.6 ± 0.9
Taste changes	49 (25)	2.1 ± 1.2	151 (77)	3.4 ± 1.5	119 (65)	2.4 ± 1.2	109 (62)	2.3 ± 1.3
Nausea	26 (13)	2.2 ± 1.0	75 (38)	2.6 ± 1.3	18 (19)	2.2 ± 0.8	24 (14)	1.8 ± 1.0
Constipation	41 (21)	1.7 ± 1.0	78 (40)	2.0 ± 1.1	36 (20)	1.7 ± 1.1	43 (24)	1.7 ± 0.9
Diarrhoea	31 (16)	1.5 ± .8	50 (25)	1.8 ± 1.0	31 (17)	1.7 ± 1.1	21 (12)	1.4 ± 0.8
Smells bother me	23 (12)	1.7 ± 1.0	72 (37)	2.6 ± 1.2	41 (22)	1.9 ± 0.9	33 (19)	2.0 ± 1.0
Vomiting	6 (3)	1.8 ± 0.8	46 (23)	2.9 ± 1.4	8 (4)	2.0 ± 0.8	12 (7)	1.3 ± 0.5

### The association between clinical factors and NIS and interference with oral intake

**Fig. 1:**
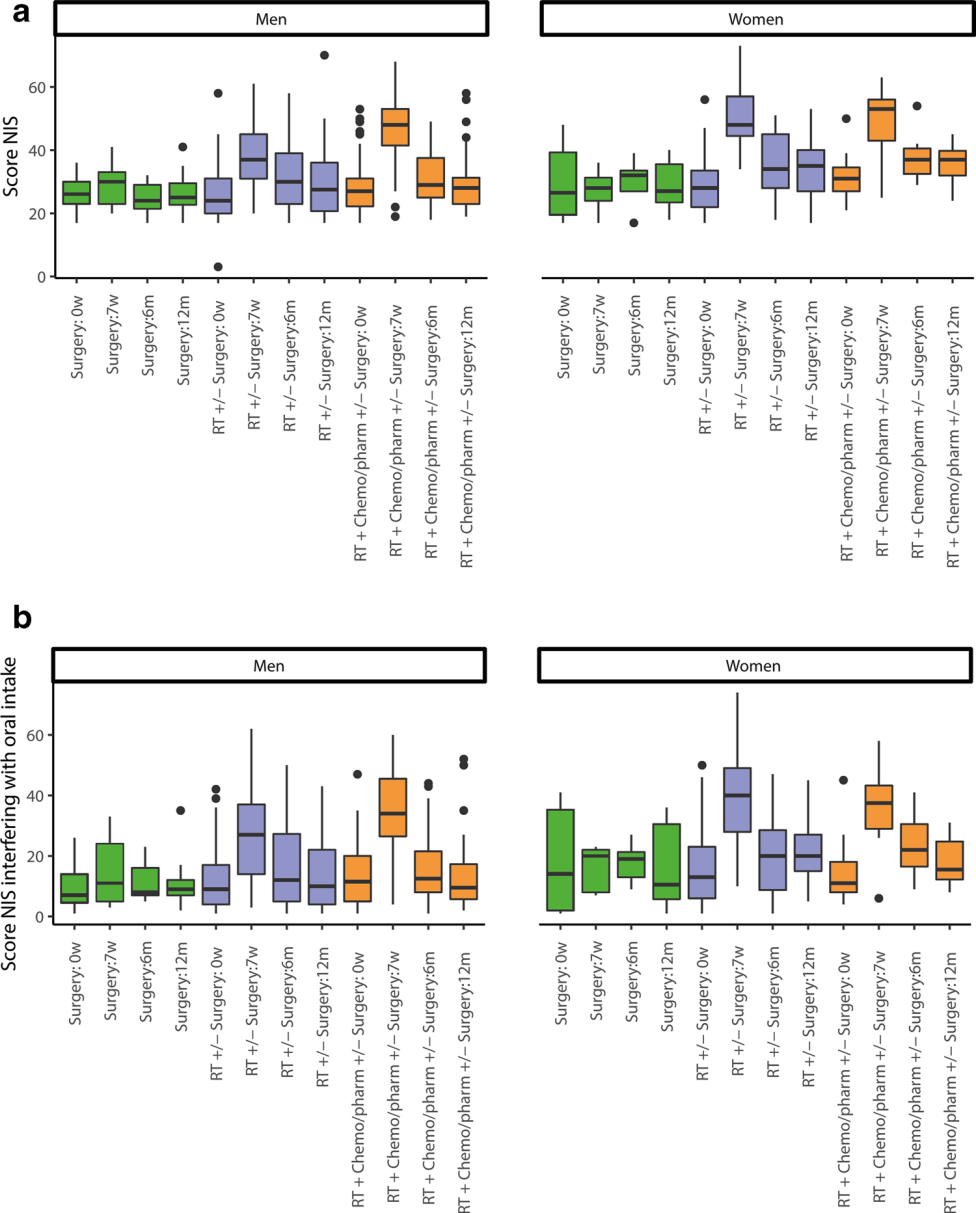
**a** Boxplot of experienced nutritional impact symptom (NIS), sum of total scores 1–5 in 17 items using the Head and Neck Patient Symptom Checklist^©^ (HNSC^©^), in men and women with different treatment approaches at baseline (0w), 7 weeks after start of treatment and at 6 and 12 months after the completion of treatment in patients with head and neck cancer. RT = radiotherapy; Chemo/pharm = chemotherapy or other pharmacological treatment. **b** Boxplot of experienced nutritional impact symptom (NIS) interfering with oral intake as the sum of total scores 1–5 on 17 items using the Head and Neck Patient Symptom Checklist^©^ (HNSC^©^), in men and women with different treatment approaches at baseline (0w), 7 weeks after the start of treatment and at 6 and 12 months after the completion of treatment in patients with head and neck cancer. RT = radiotherapy; Chemo/pharm = chemotherapy or other pharmacological treatment

At the 7-week follow-up, NIS and NIS interfering with oral intake were significantly associated with being female, being treated with RT  ± surgery, RT  + chemotherapy, or other pharmacological treatment ± surgery, and having oropharyngeal or oral cancer. At the 6-month follow-up, these clinical factors remained, but also stage III + IV, associated with NIS, although only clinical stages III and IV were associated with NIS interfering with oral intake. At the 12-month follow-up, no clinical factors were associated with NIS; however, being female indicated a significant association with NIS interfering with oral intake (Table 4). Figure 1a and b illustrate the NIS and NIS interfering with oral intake in men and women and the treatment approaches at each follow-up.

**Table 4:** Association between clinical variable and nutritional impact symptoms (NIS) and NIS interfering with oral intake, sum of total scores 1–5 in 17 items using the Head and Neck Patient Symptom Checklist^©^ (HNSC^©^), and clinical variables at each follow-up in patients with head and neck cancer

	Follow-up at 7 weeks after start of treatment			Follow-up at 6 months after completion of treatment			Follow-up at 12 months after completion of treatment		
	Estimate	Lower–upper (2.5–97.5%)	*p*-value	Estimate	Lower –upper (2.5–97.5%)	*p*-value	Estimate	Lower–upper (2.5–97.5%)	*p*-value
**NIS**									
(Intercept)†	25.38	13.69–37.07	< 0.001	21.72	10.92–32.51	< 0.001	23.15	12.20–34.11	< 0.001
Female*	6.68	3.07–10.29	< 0.001	3.56	0.16–6.96	0.040	3.23	− 0.39–6.84	0.080
Age (continuous)	− 0.09	− 0.23–0.06	0.242	− 0.02	− 0.15–0.11	0.766	0.03	− 0.16–0.11	0.713
Stage III + IV†	0.67	− 2.86–4.21	0.708	3.75	0.55–6.95	0.022	2.93	− 0.42–6.28	0.086
Radiotherapy ± surgery‡	14.22	9.02–19.41	< 0.001	5.67	0.86–10.47	0.021	3.34	− 1.35–8.04	0.161
Radiotherapy + chemotherapy or other pharmacological treatment ± surgery‡	19.30	13.12–25.48	< 0.001	4.73	− 0.96–10.42	0.103	2.01	− 3.53–7.55	0.475
Oropharyngeal cancer ≠	8.11	3.26–12.97	0.001	4.56	0.12–8.99	0.044	4.98	− 0.02–9.99	0.051
Oral cancer ≠	8.98	3.59–14.36	0.001	6.47	1.48–11.45	0.011	5.43	− 0.17–11.02	0.057
Other ≠ ˚	1.45	− 4.53–7.42	0.633	1.20	− 4.16–6.56	0.659	3.36	− 2.57–9.29	0.265
**NIS interfering with oral intake**									
(Intercept)†	5.43	− 11.27–22.12	0.522	10.03	− 5.17–25.2	0.194	3.96	− 9.31–17.22	0.556
Female*	5.42	0.18–10.66	0.043	3.42	− 1.31–8.14	0.155	4.68	0.28–9.09	0.037
Age (continuous)	− 0.05	− 0.25–0.16	0.655	0.02	− 0.21–0.16	0.810	0.05	− 0.12–0.21	0.576
Stage III + IV†	3.41	− 1.57–8.38	0.179	6.39	1.87–10.92	0.006	3.71	− 0.35–7.78	0.073
Radiotherapy ± surgery‡	15.43	7.96–22.90	< 0.001	3.32	− 3.94–10.58	0.367	2.52	− 3.11–8.16	0.377
Radiotherapy + chemotherapy or other pharmacological treatment ± surgery‡	19.36	10.52–28.19	< 0.001	0.47	− 8.04–8.98	0.913	0.21	− 6.48–6.90	0.951
Oropharyngeal cancer ≠	12.97	5.99–19.96	< 0.001	5.00	− 1.37–11.38	0.123	5.21	− 1.19–11.60	0.110
Oral cancer ≠	13.67	5.87–21.45	0.001	4.32	− 2.82–11.46	0.233	5.06	− 2.02–12.14	0.160
Other ≠ ˚	2.35	− 6.25–10.95	0.590	− 1.47	− 9.05–6.11	0.701	1.48	− 5.94–8.90	0.694

^†^The intercept value is interpreted as the NIS/NIS interference with oral intake score of one patient with all clinical variables at reference levels. *Reference: male, †reference: stage I + II, ‡reference: surgery, ≠ reference: laryngeal cancer. ˚Hypopharyngeal cancer, cancer of unknown primary, salivary gland cancer, nasal and sinus cancer, nasopharyngeal cancer, and cancer involving the external auditory canal

Association was estimated by multivariate linear regression with all clinical variables in the same models. Estimates were interpreted as actual increases in the sum of NIS scores (range 17–85) and the sum of NIS score interfering with oral intake (range 17–85)

### Body weight

The patients’ mean body weight (kg) at baseline, at 7 weeks of follow-up, and at 6 and 12 months after treatment was 82.8 (*SD* = 17.5), 78.9 (*SD* = 16.2), 77.7 (*SD* = 15.7), and 78.5 (*SD* = 16.6), respectively. At 7 weeks, 6 months, and 12 months of follow-up, 87/197 (44%), 102/184 (55%), and 86/177 (49%), respectively, of the patients had a body weight loss of > 5% in relation to baseline weight.

**Fig. 2:**
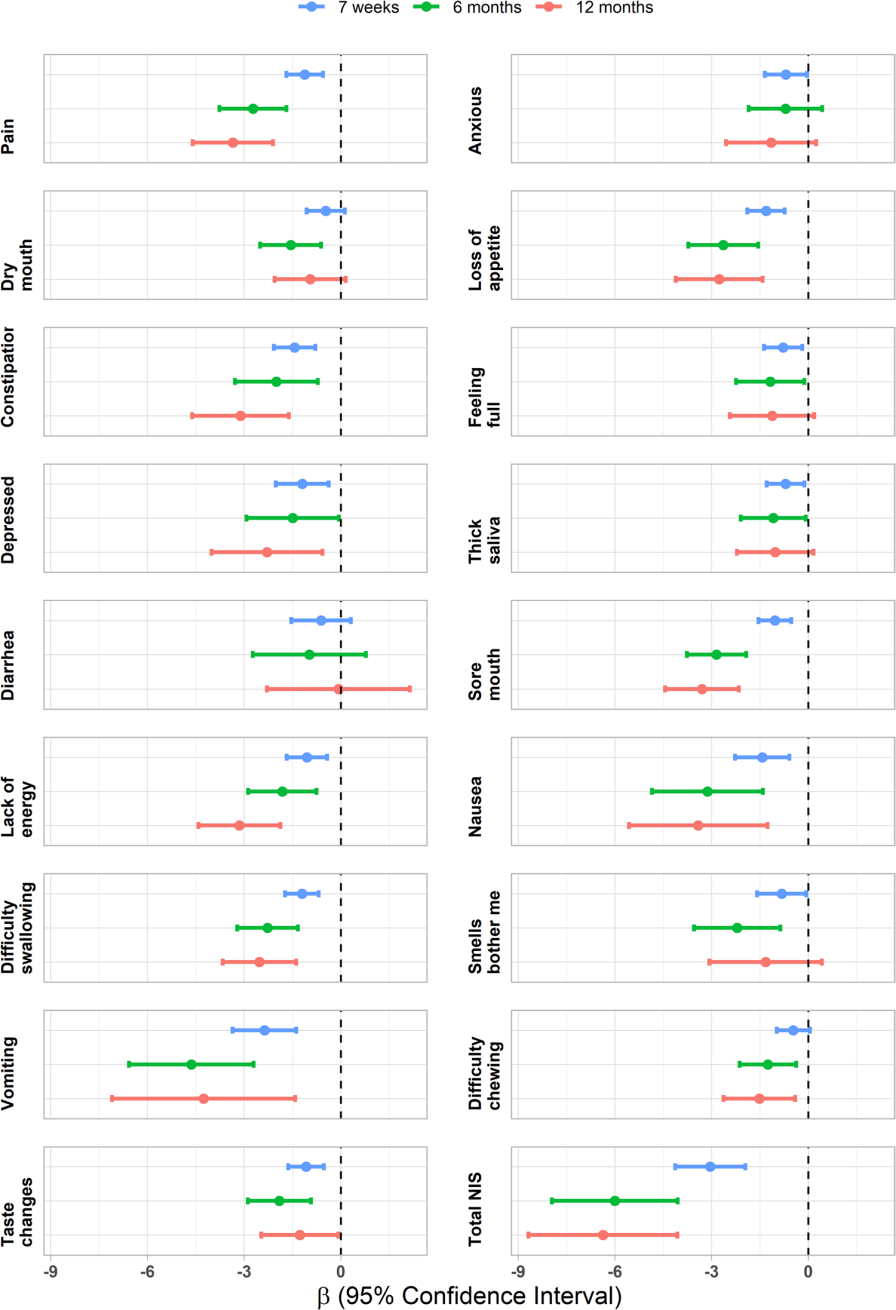
Forest plot showing the slope (β) for the effects of cumulative nutritional impact symptoms (NIS) on percentage weight loss (7 weeks, 6 and 12 months), with 95% confidence intervals. β is the expected percentage change in body weight from baseline when increasing the cumulative NIS with one point. For enabling comparisons to the individual NIS, NIS total score has here been transformed to a scale between 1 and 5 points before analysis

The MMRM models show that a higher cumulative NIS total score and cumulative NIS interfering with oral intake total score were significantly associated with greater body weight loss at all follow-ups (*p* =  < 0.001). For example, at follow-up at 7 weeks, one point increase in NIS scores, on the scale between 17 and 85, implies an expected decrease in body weight of 0.18% (95% *CI*: [0.014%, 0.24%], *p* =  < 0.001) (Table 5). The cumulative NIS and its association with changes in body weight during the study period for each of the 17 individual NIS are displayed in a forest plot (Fig. 2). The forest plot shows the following: at the 7-week follow-up, all cumulative NIS *except* dry mouth, diarrhoea, and chewing difficulties were significantly associated with changes in body weight; at follow-up 6 months all, cumulative NIS *except* anxious and diarrhoea; and finally at follow-up, 12 months all cumulative NIS *except* anxious, dry mouth, feeling full, thick saliva, diarrhoea, and smells bother me were significantly associated with changes in body weight.

**Table 5:** The association between percentage weight loss and cumulative nutritional impact symptoms (NIS) and cumulative NIS interfering with oral intake at each follow-up. β is the — slope in association, as estimated from marginal trends derived from a mixed model for repeated measures. The value of β represents the expected change in percentage weight loss when increasing one point on the total NIS scale (range 17 to 85)

	*β*	95% *CI*		*p*-value
		Lower	Upper	
NIS score				
Follow-up 7 weeks	− 0.179	− 0.243	− 0.014	< 0.001
Follow-up 6 months	− 0.353	− 0.486	− 0.238	< 0.001
Follow-up 12 months	− 0.375	− 0.511	− 0.239	< 0.001
NIS interfering with oral intake score				
Follow-up 7 weeks	− 0.126	− 0.174	− 0.0773	< 0.001
Follow-up 6 months	− 0.242	− 0.331	− 0.153	< 0.001
Follow-up 12 months	− 0.266	− 0.373	− 0.1595	< 0.001

### Body weight loss ≥ 10% at 12 months after treatment

Patients were divided into two groups according to weight loss of < 10% and ≥ 10%. At the 12-month follow-up, 46/177 (26%) of the patients had a ≥ 10% body weight loss in relation to baseline weight. After adjusting for the clinical parameters, a logistic regression analysis was used. For every unit of increase in NIS score for the ≥ 10% weight loss group at 12 months, the odds ratio increased by 8% (95% *CI*: 1.03–1.12, *p* = 0.001), and for NIS interfering with oral intake, the increase was 6% (95% *CI*: 1.02–1.10, *p* = 0.002).

The univariate analysis using Pearson’s chi-squared or Fisher’s exact test identified the following NIS to be significantly associated with ≥ 10% weight loss: pain (*p* = 0.002), loss of appetite (*p* = 0.002), feeling full (*p* = 0.003), sore mouth (*p* = < 0.001), difficulty swallowing (*p* = 0.001), and taste changes (*p* = 0.004). NIS interfering with oral intake that were significantly associated with ≥ 10% weight loss were pain (*p* = < 0.001), loss of appetite (*p* = < 0.001), feeling full (*p* = < 0.001), sore mouth (*p* = < 0.001), difficulty swallowing (*p* = 0.001), taste changes (*p* = < 0.001), and dry mouth (*p* = 0.026).

## Discussion

This observational study demonstrates that patients had the greatest symptom and oral intake interference burden at the 7-week follow-up. One year after treatment, it was NIS swallowing and chewing difficulties, and loss of appetite that mainly interfered with oral intake in patients with HNC. At 12 months after treatment, NIS scores still had not returned to baseline, while the scores of NIS interfering with oral intake had. During the 12-month study period, the total cumulative NIS and cumulative NIS interfering with oral intake were associated with body weight. NIS and NIS interfering with oral intake (taste changes, pain, loss of appetite, feeling full, sore mouth, swallowing difficulties, and in interfering, dry mouth) increased the risk for a body weight loss of ≥ 10% 1 year after treatment.

There are several instruments to assess HRQoL in patients with HNC, and the HNSC^©^ is a questionnaire constructed to map symptoms that may affect nutritional intake. By using the HNSC^©^, the changes in experienced NIS and NIS interfering with oral intake over 1 year after treatment were visualized in a large mixed cohort of patients with HNC. The fact that several NIS were present at baseline before the initiation of treatment means that these NIS can foremost be interpreted as tumour-related symptoms such as pain, anxiety, and sore mouth. The NIS at the subsequent time points shifted to more treatment-related and long-lasting symptoms such as dry mouth, thick saliva, and taste changes. The NIS scores at follow-up 12 months are in line with a review by Höxbro et al. (2017); however, the previous research does not provide information which symptoms that interfere with oral intake [29]. To different degrees, all 17 NIS were presented at baseline, and this is in line with previous research reporting several NIS being present before the start of treatment [30]. The symptom burden peaking at 7-week follow-up, which was for the majority at the termination of treatment, is also described in the earlier research [31]. Lack of energy, anxiety, and depression occurred to different degrees at the follow-ups in the present study, and except for the 7-week follow-up, these symptoms were not considered to strongly affect oral intake, which is in line with Jin et al. [23]. These factors need to be continuously monitored by health care professionals because they may occur at any time from around the time of diagnosis to beyond the end of treatment.

In the present study, females scored higher compared to men in both NIS and NIS interfering with oral intake at all follow-ups. This result is interesting because gender differences are rarely reported in research on HNC; however, some existing research does report differences in experienced HRQoL between men and women with HNC [32] and suggests that women tend to experience acute and severe side effects to a greater extent [33]. The present results call for further research focusing on gender effects in patients with HNC, which is numerically dominated by men, thus putting female voices at risk of being diminished. Advanced stage is reported to be associated with decreased HRQoL even at 12 months after treatment [17, 34]. These earlier findings are not in line with the present results, where no clinical factors remained associated with NIS at 12 months. However, the present result for clinical stage might not be comparable with earlier research because in the present study stage was classified according to the Union for International Cancer Control (UICC) 8th edition, thus downscaling the stage in human papilloma virus-positive oropharyngeal cancer. Patients with oropharyngeal or oral cancer are, due to the tumour location and treatment, reported to be at risk for long-term difficulties related to eating, such as saliva issues and swallowing and chewing difficulties [29]. It should be emphasized that in the present study neither oropharyngeal nor oral cancer remained significantly associated with NIS at 12 months or significantly associated with NIS interfering with oral intake at 6 or 12 months of follow-up. Already at the 6 months follow-up, the number of clinical factors affecting NIS interfering with oral intake had decreased, suggesting that the patients had, at least to some degree, adapted to new eating strategies [35], but also, as found by Ganzer et al. (2015) that difficulties may be “downplayed” [36] and become “the new normal”. One can also speculate that newer treatment options are associated with fewer side effects.

The weight curve for the study population displayed a nadir at the 6-month follow-up, which is in line with previous research [1, 7, 8]. At the 7-week follow-up, 44% of the patients had a weight loss ˃ 5% in relation to baseline weight, which was a lower number compared to Jin et al. (2021), who reported as much as 72% at the end of treatment, a finding that may be explained by more patients receiving concurrent chemotherapy in that study [23]. During the study period, the number of patients with weight loss ˃ 5% increased to 55% and 49% at 6 and 12 months of follow-up, respectively. According to Einarsson et al. (2020), a body weight loss of ˃ 5% during the last 6 months and a C-reactive protein > 5 mg/L are useful parameters in diagnosing malnutrition in patients with HNC [37]. Although no attention was given to C-reactive protein measurements in the present study, the number of patients with a weight loss of ˃ 5% may indicate that about half of the population was at risk for malnutrition. At all 3 follow-ups, cumulative NIS and cumulative NIS interfering with oral intake were contributing to weight loss, and the forest plot indicate several single NIS to be associated with changes in body weight at all 3 follow-ups. This long-lasting NIS and NIS interfering with oral intake association with body weight is important information for health care since this highlights the need for regular follow-ups concerning nutritional intake and body weight in the long perspective.

The univariate analysis at 12 months after treatment showed that well-known symptoms (such as pain, loss of appetite, feeling full, sore mouth, difficulty swallowing, taste changes, and dry mouth) were affecting eating and increasing the risk for a body weight loss of ≥ 10%. Petrusson et al. (2005) described similar findings and reported lower HRQoL in patients with a weight loss of ≥ 10% compared to patients with less weight loss [38]. Because malnutrition is a significant problem for many patients with HNC, the findings in the present study are important in that they give further support that reduced oral intake symptoms causing weight loss are an important consideration for health care professionals treating patients with HNC also in a long-term perspective. The present study indicates that the HNSC^©^ is explicit in identifying factors affecting eating and thereby is a useful tool for nutritional surveillance of patients with HNC.

Some limitations to this study have to be considered. The design of the study that only included patients with WHO PS 0–2 may have led to a bias because patients with worse performance status can be expected to have more nutritional problems. Also, there were some missing data because some patients did not answer the HNSC^©^ at all the follow-ups. A strength of this study lies in the relatively large sample size and in the fact that this was a real-world study with a longitudinal design.

## Conclusion

The HNSC^©^ was demonstrated to be a useful tool for identifying symptom burden associated with body weight loss in patients with HNC. The greatest NIS and oral intake interference burden were found at the 7-week follow-up. Twelve months after treatment, swallowing and chewing difficulties and loss of appetite were the highest scored NIS to interfering with oral intake. Cumulative NIS total score and cumulative NIS interfering with oral intake total score were associated with greater body weight loss at all 3 follow-ups, and 12 months after termination of treatment high NIS and NIS interfering with oral intake scores were found in the group of patients with the greatest body weight loss. The HNSC^©^ is concluded to identify patients who experience NIS and NIS interfering with oral intake and may be used in clinical settings as a complement together with an individual care plan. This study shows the importance for health care professionals to take a holistic approach when meeting the patients’ nutritional needs with possible gender differences in experienced symptom burden and degree of body weight loss.

## Data Availability

N/A.

## Code availability

N/A.
